# Emergence of High Antimicrobial Resistance among Critically Ill Patients with Hospital-Acquired Infections in a Tertiary Care Hospital

**DOI:** 10.3390/medicina58111597

**Published:** 2022-11-04

**Authors:** Ahmed E. Abou Warda, Fatma Molham, Heba F. Salem, Gomaa Mostafa-Hedeab, Bashayer F. ALruwaili, Ayman N. Moharram, Mohamed Sebak, Rania M. Sarhan

**Affiliations:** 1 Clinical Pharmacy Department, Faculty of Pharmacy, October 6 University, Giza 12585, Egypt; 2 Microbiology and Immunology Department, Faculty of Pharmacy, Beni-Suef University, Beni-Suef 62514, Egypt; 3 Pharmaceutics and Industrial Pharmacy Department, Faculty of Pharmacy, Beni-Suef University, Beni-Suef 62514, Egypt; 4 Pharmacology Department, Medical College, Jouf University, Sakaka 72388, Saudi Arabia; 5 Community and Family Medicine Department, Division of Family Medicine, Medical College, Jouf University, Sakaka 72388, Saudi Arabia; 6 Critical Care Medicine Department, Faculty of Medicine, Cairo University, Giza 12613, Egypt; 7 Clinical Pharmacy Department, Faculty of Pharmacy, Beni-Suef University, Beni-Suef 62514, Egypt

**Keywords:** antibiotics, antimicrobial resistance, antimicrobial susceptibility, critically ill patients, nosocomial infections, hospital-acquired infections

## Abstract

*Background and Objectives*: Inappropriate antibiotic usage in hospitalized patients contributes to microbial resistance. Our study aimed to examine the incidence of clinical bacterial isolates and their antibiotic resistance burden among critically ill patients in different hospital units. *Materials and Methods*: A single-centered cross-sectional study was conducted in a 120-bed tertiary care hospital that included 221 critically ill patients with hospital-acquired infections. Bacterial cultures and sensitivity reports were obtained and followed by a formal analysis of the antibiogram results to explore recovered isolates’ prevalence and antibiotic susceptibility patterns. *Results*: Gram-negative bacteria were the most predominant pathogens among recovered isolates from the various hospital units (71%). *Klebsiella* sp. was the most prevalent microbe, followed by *Acinetobacter* sp., with an incidence level of 28% and 16.2%, respectively. Among the Gram-positive organisms, the coagulase-negative *Staphylococci* were the most predominant organism (11.3%), while (6.3%) methicillin-resistant *Staphylococcus aureus* (MRSA) isolates were recovered from different hospital units. Antibiotic sensitivity testing showed that polymyxin B was the most effective antibiotic against Gram-negative bacteria, whereas vancomycin and linezolid were the most active antibiotics against Gram-positive pathogens. Moreover, 7% of the Gram-negative bacteria isolated from different units showed positive production of extended-spectrum beta-lactamase (ESBL). *Conclusions:* The current study describes the high antibiotic resistance patterns in various hospital units that need extra legislation to prevent healthcare providers from misprescription and overuse of antibiotics.

## 1. Introduction

Nosocomial infections are spreading worldwide with millions of patients affected each year [1], especially in developing countries [2,3]. Nosocomial infection is an infection developed 2 to 3 days after admission to any healthcare unit or during a maximum of 3 days after leaving the unit in a condition that the patient was not infected with the same microbe on first admission [4]. Various species of Gram-negative and Gram-positive bacteria are causing nosocomial infections according to several epidemiological analyses [5].

The intensive care unit (ICU) is one of the leading units among healthcare settings in the percentage of the population with nosocomial infections [6], because of its patients who are susceptible to various infections due to immunodeficiency, utilization of invasive medical equipment, administration of various medications, or deformation of normal flora [7,8]. According to the Extended Prevalence of Infection in Intensive Care (EPIC) II, Gram-negative bacteria including *Acinetobacter* sp., *Pseudomonas* sp., *Escherichia coli* (*E. coli*), *Klebsiella* sp., and *Enterobacter* sp. had the highest overall predominance in the ICU, whereas *Staphylococcus aureus* (*S. aureus*) was the most predominant microbe among recovered microorganisms [9]. In addition, the high susceptibility of patients in the ICU to microbial infection leads to an increase in the chance of antibiotic resistance among ICU-derived pathogens [7,10].

The excessive use of antimicrobial agents in different healthcare units to combat various infectious diseases is one of the main causes of the emergence of antibiotic resistance in hospitals, leading to more and more nosocomial infections among patients [11,12]. Notably, the COVID-19 pandemic has recently caused a surge in antibiotic resistance, which is one of the most serious health issues affecting individuals worldwide [13]. Self-medication with antibiotics, empirical antibiotic therapy, and antibiotics prescribed by general practitioners were all risk factors for elevated antibiotic resistance levels during the COVID-19 outbreak [14]. In low- and middle-income nations with weak antibiotic control regimes, access to antibiotics without a prescription is directly impacted by both anxiety and improper antibiotic use [15]. Additionally, during the COVID-19 epidemic, the use of biocides greatly expanded on a global scale. The amount of indirect pressure that caused antibiotic resistance was probably increased by these biocides [16].

The predominance of multidrug-resistant (MDR) microorganisms that gain resistance to multiple antibiotics through inherent or acquired mechanisms restricts the choice of antimicrobials in their treatment, leading to a long stay of patients in hospitals and increased healthcare costs [3,17,18]. Thus, it is necessary to determine the prevalence of microbial pathogens and their antibiotic susceptibility patterns to employ an effective therapeutic strategy to combat their spread among patients, especially in healthcare units [3,19,20,21].

It is noteworthy that epidemiological studies investigating the prevalence of microbial pathogens and their resistance pattern are running day after day to help to manage the quick spread of microbial infections with multidrug-resistant bacteria. However, comprehensive updates on the prevalence of microbial pathogens with their susceptibility to different drugs in all healthcare locations are not available [22]. In addition, the microbial species among hospitals vary over time within different hospitals in the same country and even within various units in the same hospital. Furthermore, microbial resistance to antimicrobials is dynamic, with great changes taking place over time in antibiotics sensitivity patterns [3]. Herein, we aimed to examine the frequency of the various bacterial pathogens, including Gram-positive and Gram-negative bacteria, recovered from clinical specimens collected from patients in different hospital units in a tertiary care hospital. Moreover, our study focused on the susceptibility patterns of different microbes to various antibiotics to estimate antibiotic resistance burden and determine the best therapeutic agents available to fight against recovered microorganisms.

## 2. Materials and Methods

### 2.1. Study Design and Patients

This was a single-centered cross-sectional study conducted on a 120-bed tertiary care hospital in Cairo, Egypt, between January and July 2021. The study was approved by the Ethics Committee and Institutional Review Board of the Faculty of Pharmacy, Beni-Suef University, with approval number REC-H-PhBSU-22003, 16 February 2021. The study was carried out following the Helsinki Declaration’s principles.

Inclusion criteria included adult patients selected by non-random consecutive sampling technique who were aged >18 years and diagnosed with at least one hospital-acquired infection after 48 h of admissions, such as hospital-acquired pneumonia (HAP), ventilator-associated pneumonia (VAP), urinary tract infections (UTIs), bloodstream infections (BSIs), catheter-related bloodstream infections (CRBSIs), intra-abdominal infections (IAIs), and skin and skin soft tissue infections (SSTIs), within critical care units of the hospital including the general intensive care unit (ICU), intermediate care unit (INT), cardiac care unit (CCU), open-heart surgery unit (OH), and general surgery unit (SUR).

Exclusion criteria included outpatients, pregnancy, lactation, and a patient who started empirical antibiotics before collecting specimens or patients with evidence of infection at the time of hospital admission.

### 2.2. Isolation and Identification of the Clinical Isolates

Clinical samples of recruited patients (sputum, urine, wound, pus, and blood) were collected in sterile containers followed by isolation and identification of microorganisms from the specimens according to the standard protocols in the laboratory.

Aerobic culture media were used to inoculate the blood samples, while the rest of the clinical specimens were inoculated onto blood agar and cysteine lactose electrolyte deficient (CLED) using a calibrated wire loop (0.001 mL) and then incubated overnight at 37 °C. Gram-positive bacteria were cultured and identified using blood agar due to their hemolytic activity, while Gram-negative bacteria were cultured and identified using CLED based on their lactose fermentation capabilities. Significant colony counts were determined as those over 10^5^ colony-forming units per milliliter (CFU/mL). Culture plates that exhibited no bacterial growth were incubated for a further 48 h.

The confirmation of bacterial identification was accomplished through Gram staining, morphological characterization, and different biochemical tests depending on the type of re-covered microorganisms, such as the catalase reaction, slide and tube coagulase tests, culture on DNase agar, and bile esculin for identification of Gram-positive bacteria, whereas biochemical tests such as oxidase, triple sugar iron, motility indole ornithine, citrate, lysine iron arginine, and urease tests were used to identify Gram-negative bacteria.

### 2.3. Antimicrobial Susceptibility Testing

All recovered microorganisms were tested for antibiotic susceptibility using the Kirby–Bauer disc diffusion method on Mueller–Hinton agar, and the findings were interpreted according to the Clinical and Laboratory Standard Institute (CLSI) guidelines [23]. The diameter of the zones of inhibition for each antibiotic was measured and interpreted as resistant, intermediate, or susceptible as pre-described in CLSI guidelines [23]. The choice of the tested antibiotics was based on the available drugs that were frequently prescribed in the hospital and on the spectrum of activity of the tested antimicrobials on Gram-positive and Gram-negative bacteria, as illustrated in Table S1.

Further, the clinical isolates of the Gram-positive *S*. *aureus*, which were cefoxitin-resistant, were assigned as MRSA as recommended by CSLI [23] and other previous reports [24]. Moreover, the production of ESBL in Gram-negative organisms was detected via the double-disc synergy test on Mueller–Hinton agar media according to the CLSI guidelines [23].

### 2.4. Statistical Analysis

The patients’ collected specimens and isolated bacterial strains, along with antibiotic sensitivity profiles were registered in the WHONET 5.6 software (WHO, Geneva, Switzerland), a windows-based database for the management and analysis of microbiology laboratory data with a particular focus on antimicrobial resistance surveillance, developed and supported by the WHO Collaborating Centre for Surveillance of Antimicrobial Resistance at the Brigham and Women’s Hospital in Boston, MA, USA [25]. Categorical data are presented as numbers and proportions. Normality testing was conducted using the Shapiro–Walk test. For non-normally distributed continuous data, the median and interquartile range was used. Statistical analyses were carried out using the Statistical Package for Social Sciences (SPSS), version 26 (SPSS Inc., Chicago, IL, USA). All graphs and charts in the study were prepared using EXCEL, (Microsoft Excel 2016 64-Bit Edition, Redmond, WA, USA) whereas the heatmaps were generated using the R-statistical platform (version x 64, 4.1.2, Toulouse, France) [26].

## 3. Results

### 3.1. Demographic Data of Included Patients

**Figure 1:**
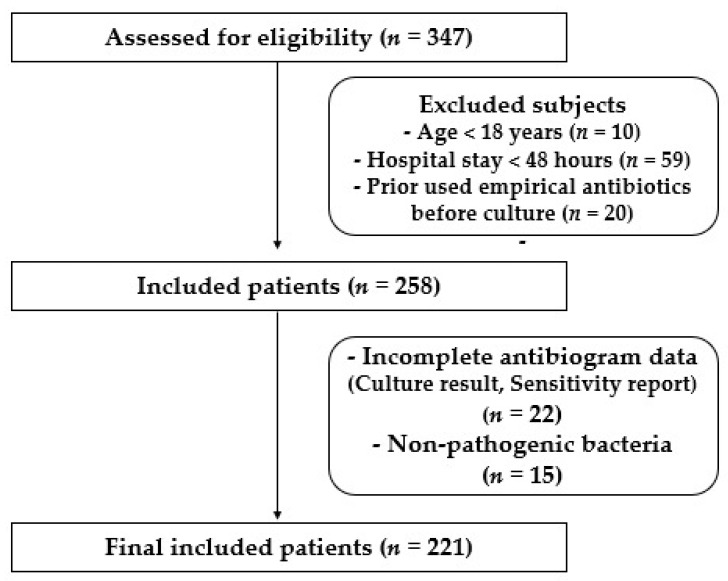
Flow chart display recruited patients in the study.

A total of 347 patients were assessed for eligibility; 111 patients did not meet inclusion criteria, while 221 patients with clinical evidence of hospital-acquired infection (HAP, VAP, BSI, CRBSI, IAI, UTI, and SSTI) were enrolled in the study as shown in Figure 1. Among the enrolled subjects, 156 patients (70.6%) were male. The majority (57.5%) of patients were found in the age group of >60 years with a median age of 65 years. Table 1 depicts the demographic characteristics of the patients.

### 3.2. Prevalence of the Bacterial Isolates in Different Hospital Units

**Figure 2:**
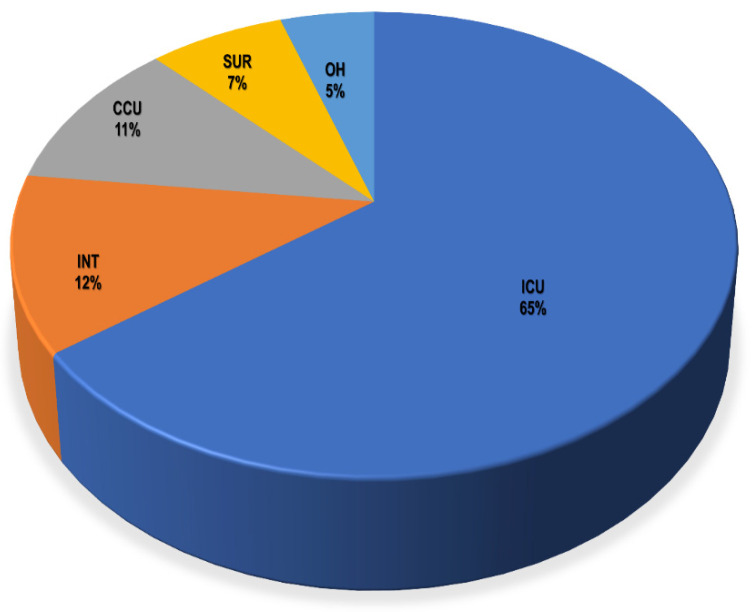
Percentage of total recovered bacterial isolates from different hospital units. ICU: intensive care unit, INT: intermediate care unit, CCU: cardiac care unit, OH: open-heart surgery unit, SUR: general surgery unit.

Clinical specimens from recruited patients in the ICU, CCU, INT, SUR, and OH yielded a total of 221 clinical isolates for testing. A total of 157 Gram-negative bacteria and 64 Gram-positive bacteria were isolated from different units (Table S2). The ICU had the biggest share of total isolates with 143 isolates (64.7%), followed by the INT from which 27 bacterial isolates (12.2%) were recovered, whereas the smallest number of recovered isolates was from the OH, with only 11 recovered microorganisms (5%), as shown in Figure 2 and Table S2. Regarding the recovered isolates, the Gram-negative bacteria *Klebsiella* sp. were the most predominant bacteria among them, with a total of 62 isolates, as presented in Table S3 and Figure S1. Next to *Klebsiella* sp., *Acinetobacter* sp., and *Pseudomonas* sp. as well as the Gram-positive bacteria, coagulase-negative *Staphylococcus* had total numbers of 36, 31, and 25, respectively.

**Figure 3:**
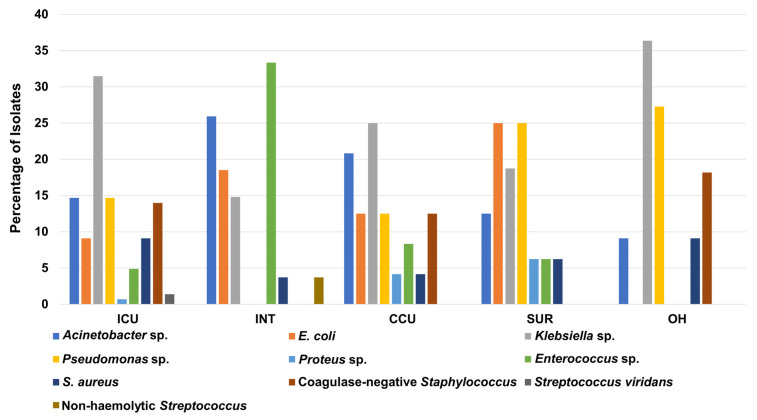
Percentage of recovered Gram-positive and Gram-negative bacteria from patients in different hospital units. ICU: intensive care unit, INT: intermediate care unit, CCU: cardiac care unit, OH: open-heart surgery unit, SUR: general surgery unit.

Similarly, *Klebsiella* sp. had the highest percentage of total isolates in the ICU, CCU, and OH with percentages of 31%, 25%, and 36%, respectively. In contrast, *Acinetobacter* sp. had the highest percentage in the INT with 26%, whereas *E*. *coli* and *Pseudomonas* sp. showed the biggest share among clinical isolates recovered from the SUR, with a percentage of 25% for both (Figure 3). As expected from the previous statistics, the ICU showed the highest number of bacterial isolates compared to other units for *Acinetobacter* sp., *E*. *coli*, *Klebsiella* sp., *Pseudomonas* sp., *S. aureus*, and coagulase-negative *Staphylococcus* (Table S4).

On the other hand, extra investigation of the ICU which had the highest share of clinical isolates amongst all hospital units revealed that *Klebsiella* sp. was the most frequent microorganism among recovered ICU isolates, with a total of 45 isolates (31%), as shown in Table S4. Both *Acinetobacter* sp. and *Pseudomonas* sp. were the second most predominant microorganisms recovered from the ICU, with a total number of 21 isolates for both, followed by the Gram-positive bacteria, coagulase-negative *Staphylococcus* with a total of 20 isolates.

**Figure 4:**
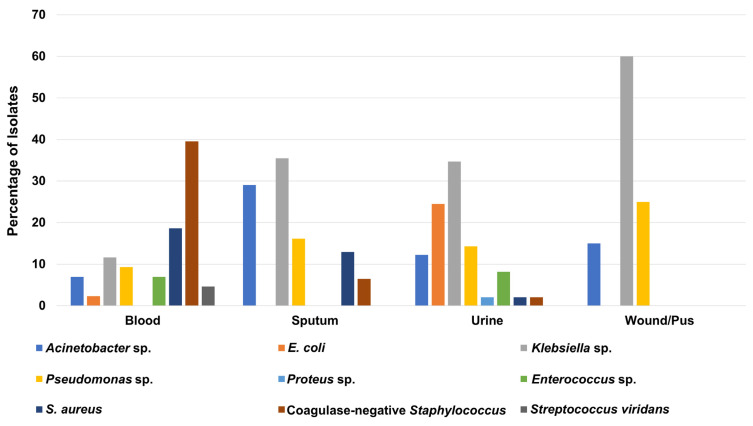
Percentage of recovered Gram-positive and Gram-negative bacteria from different clinical specimens collected from patients in the intensive care unit.

As presented in Table S5, more than one-third of ICU isolates (49 isolates; 34.3%) were recovered from urine samples, while 43 clinical isolates (30%) were isolated from blood samples. Interestingly, *Klebsiella* sp. was the most frequent microorganism in urine, pus/wound, and sputum samples, with total numbers of 17 (34.7%), 12 (60%), and 11 (35.5%) isolates, respectively (Figure 4 and Table S5). In contrast, the coagulase-negative *Staphylococcus* was the most predominant microbe among the blood samples collected from ICU patients, with a total of 17 (39.5%) isolates (Figure 4 and Table S5).

### 3.3. Antibiotic Sensitivity Pattern of Recovered Isolates from Different Hospital Units

**Figure 5:**
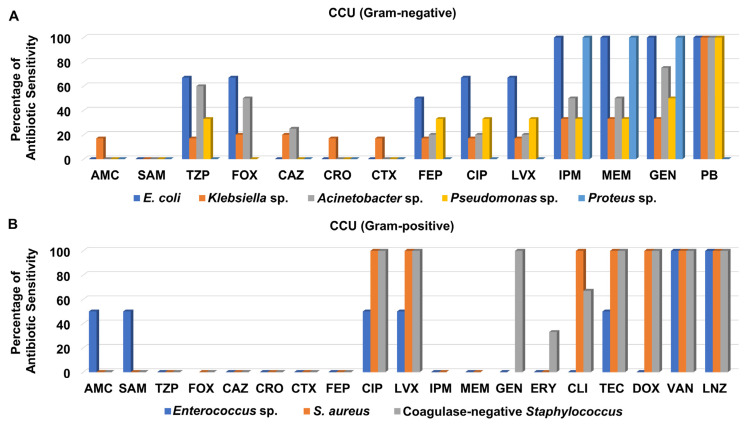
Percentage of antibiotic sensitivity of different bacterial isolates recovered from the critical cardiac unit. (**A**) Antibiotic sensitivity pattern of Gram-negative bacteria. (**B**) Antibiotic sensitivity pattern of Gram-positive bacteria. AMC: amoxicillin/clavulanate, SAM: ampicillin/sulbactam, TZP: piperacillin/tazobactam, FOX: cefoxitin, CAZ: ceftazidime, CRO: ceftriaxone, CTX: cefotaxime, FEP: cefepime, CIP: ciprofloxacin, LVX: levofloxacin, IPM: imipenem, MEM: meropenem, GEN: gentamicin, PB: polymyxin B, ERY: erythromycin, CLI: clindamycin, TEC: teicoplanin, DOX: doxycycline, VAN: vancomycin, LNZ: linezolid.

Screening of antibiotic sensitivity patterns of clinical isolates recovered from different specimens from the CCU revealed that most of the Gram-negative bacteria were not sensitive to the cephalosporins; ceftazidime, ceftriaxone, and cefotaxime as well as the aminopenicillins/beta-lactamase inhibitors; amoxicillin/clavulanate, and ampicillin/sulbactam (Figure 5A and Table S6). As presented in Figure 5A, both the carbapenems, imipenem and meropenem, and the aminoglycoside gentamicin exhibited moderate to good antibacterial activity against the Gram-negative bacteria isolated from CCU patients, especially against *E*. *coli*, *Proteus* sp. and *Acinetobacter* sp., while they showed weaker activity against *Pseudomonas* sp. and *Klebsiella* sp. It is noteworthy that polymyxin B displayed the best antimicrobial activity against all Gram-negative bacteria isolated from CCU patients except for *Proteus* sp.

On the other hand, most of the Gram-positive bacteria recovered from CCU patients were not susceptible to ceftazidime, ceftriaxone, cefotaxime, cefepime, and amoxicillin/clavulanate, ampicillin/sulbactam, piperacillin/tazobactam, and erythromycin (Figure 5B and Table S6). Only one isolate of *S*. *aureus* was recovered from CCU patients and assigned as MRSA. The clinical isolates of MRSA, and coagulase-negative *Staphylococcus* were sensitive to both levofloxacin and ciprofloxacin, whereas neither *Enterococcus* sp. nor MRSA were susceptible to either imipenem or meropenem. Interestingly, the isolates of MRSA and coagulase-negative *Staphylococcus* were sensitive to glycylcycline, teicoplanin, and tetracycline, doxycycline, whereas all CCU Gram-positive bacteria were susceptible to the glycopeptide vancomycin and the oxazolidinone linezolid (Figure 5B and Table S6).

**Figure 6:**
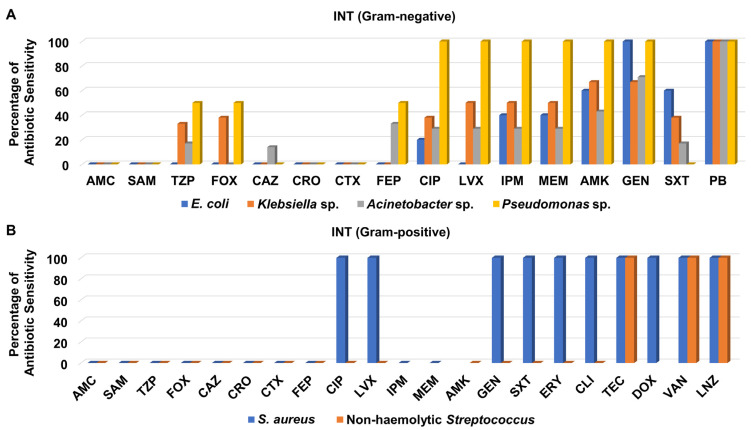
Percentage of antibiotic sensitivity of different bacterial isolates recovered from the intermediate care unit. (**A**) Antibiotic sensitivity pattern of Gram-negative bacteria. (**B**) Antibiotic sensitivity pattern of Gram-positive bacteria. AMC: amoxicillin/clavulanate, SAM: ampicillin/sulbactam, TZP: piperacillin/tazobactam, FOX: cefoxitin, CAZ: ceftazidime, CRO: ceftriaxone, CTX: cefotaxime, FEP: cefepime, CIP: ciprofloxacin, LVX: levofloxacin, IPM: imipenem, MEM: meropenem, GEN: gentamicin, PB: polymyxin B, ERY: erythromycin, CLI: clindamycin, TEC: teicoplanin, DOX: doxycycline, VAN: vancomycin, LNZ: linezolid.

Like the CCU isolates, the Gram-negative bacteria recovered from the INT were not susceptible to ceftazidime, ceftriaxone, cefotaxime, amoxicillin/clavulanate, and ampicillin/sulbactam except for one *Acinetobacter* sp. isolate which was sensitive to ceftazidime (Figure 6A and Table S7). It is noteworthy that the aminoglycosides amikacin and gentamicin showed good antibacterial activity against most of the Gram-negatives isolates derived from INT patients, while polymyxin B was the best antibacterial agent evaluated with 100% sensitivity of all of them (Figure 6A).

Although only one isolate of *S*. *aureus* was recovered from INT patients, it was recorded as MRSA. MRSA and non-hemolytic *Streptococcus* isolates recovered from the INT did not show any sensitivity to cefoxitin, ceftazidime, ceftriaxone, cefotaxime, cefepime, amoxicillin/clavulanate, ampicillin/sulbactam, and piperacillin/tazobactam (Figure 6B and Table S7). Notably, these isolates were totally sensitive to teicoplanin, vancomycin, and linezolid, as shown in Figure 6B.

**Figure 7:**
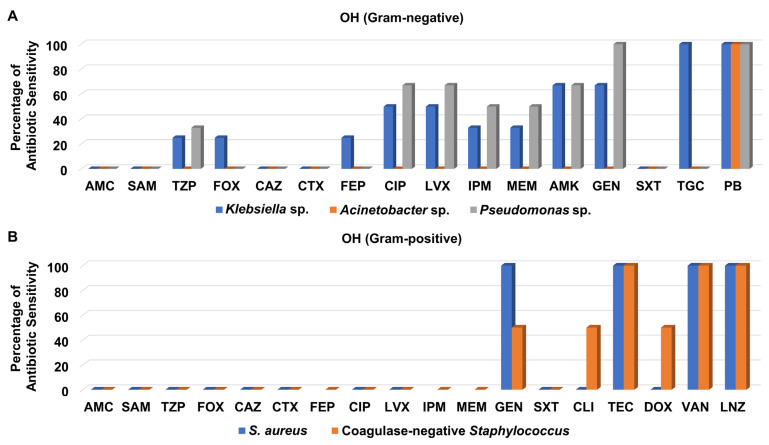
Percentage of antibiotic sensitivity of different bacterial isolates recovered from the open-heart surgery unit. (**A**) Antibiotic sensitivity pattern of Gram-negative bacteria. (**B**) Antibiotic sensitivity pattern of Gram-positive bacteria. AMC: amoxicillin/clavulanate, SAM: ampicillin/sulbactam, TZP: piperacillin/tazobactam, FOX: cefoxitin, CAZ: ceftazidime, CRO: ceftriaxone, CTX: cefotaxime, FEP: cefepime, CIP: ciprofloxacin, LVX: levofloxacin, IPM: imipenem, MEM: meropenem, GEN: gentamicin, PB: polymyxin B, ERY: erythromycin, CLI: clindamycin, TEC: teicoplanin, DOX: doxycycline, VAN: vancomycin, LNZ: linezolid.

Regarding the Gram-negative bacteria isolated from the OH, they were not susceptible to amoxicillin/clavulanate, ampicillin/sulbactam, ceftazidime, cefotaxime, and trimethoprim/sulfamethoxazole (Figure 7A and Table S8). Notably, *Klebsiella* sp. and *Pseudomonas* sp. clinical isolates showed moderate to high sensitivity patterns to levofloxacin, ciprofloxacin, imipenem, meropenem, amikacin, and gentamicin, whereas *Acinetobacter* sp. isolate was not susceptible to any one of them. It is noteworthy that only *Klebsiella* sp. isolates were sensitive to tigecycline, while all Gram-negative bacteria isolated from the OH were susceptible to polymyxin B (Figure 7A and Table S8).

Only three Gram-positive bacteria were recovered from the OH, from which one *S*. *aureus* isolate was assigned as MRSA; they were not sensitive to amoxicillin/clavulanate, ampicillin/sulbactam, piperacillin/tazobactam, cefoxitin, ceftazidime, cefotaxime, levofloxacin, ciprofloxacin, and trimethoprim/sulfamethoxazole (Figure 7A and Table S8). However, they were all susceptible to teicoplanin, vancomycin, and linezolid, as presented in Figure 7B.

**Figure 8:**
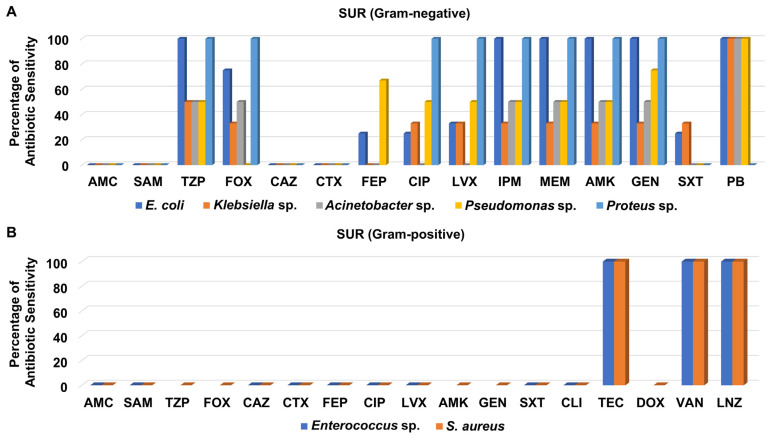
Percentage of antibiotic sensitivity of different bacterial isolates recovered from the general surgery unit. (**A**) Antibiotic sensitivity pattern of Gram-negative bacteria. (**B**) Antibiotic sensitivity pattern of Gram-positive bacteria. AMC: amoxicillin/clavulanate, SAM: ampicillin/sulbactam, TZP: piperacillin/tazobactam, FOX: cefoxitin, CAZ: ceftazidime, CRO: ceftriaxone, CTX: cefotaxime, FEP: cefepime, CIP: ciprofloxacin, LVX: levofloxacin, IPM: imipenem, MEM: meropenem, GEN: gentamicin, PB: polymyxin B, ERY: erythromycin, CLI: clindamycin, TEC: teicoplanin, DOX: doxycycline, VAN: vancomycin, LNZ: linezolid.

In contrast, the susceptibility of the Gram-negative bacteria isolated from the SUR to different antibiotics was somewhat better than that of isolates from the OH (Figure 8A). All tested Gram-negative isolates recovered from the SUR displayed moderate to high susceptibility to piperacillin/tazobactam, imipenem, meropenem, amikacin, and gentamicin (Figure 8A and Table S9). Like in other units, polymyxin B was the best antibiotic against various SUR-derived Gram-negative bacteria.

On the other hand, the two Gram-positive isolates recovered from the SUR, which were MRSA and *Enterococcus* sp., were not susceptible to most of the tested antibacterial agents, including amoxicillin/clavulanate, ampicillin/sulbactam, ceftazidime, cefotaxime, cefepime, levofloxacin, ciprofloxacin, and clindamycin, as shown in Figure 8B and Table S9. By contrast, both isolates were only sensitive to teicoplanin, vancomycin, and linezolid.

Interestingly, the Gram-negative bacteria isolated from ICU patients displayed no or weak susceptibility patterns to many tested antibiotics such as ampicillin/sulbactam, amoxicillin/clavulanate, ceftazidime, cefotaxime, ceftriaxone, and cefepime (Table S10 and Figures S2–S7). Notably, many of them displayed moderate to high susceptibility to the aminoglycosides amikacin and gentamicin, as well as the carbapenems imipenem and meropenem. Like other Gram-negative microorganisms in our study, the highest sensitivity pattern of the ICU’s Gram-negative isolates was recorded for polymyxin B (Figure S2).

Investigation of the antibiotic susceptibility patterns of the most predominant bacterial isolates in the ICU, which were *Klebsiella* sp., revealed that most of them did not show susceptibility to ampicillin/sulbactam, amoxicillin/clavulanate, ceftazidime, ceftriaxone, cefotaxime, cefepime, and trimethoprim/sulfamethoxazole (Table S10 and Figure S3). Markedly, their susceptibility to imipenem, meropenem, amikacin, and gentamicin was better than that of other previously mentioned antibiotics, whereas polymyxin B was the most active antibacterial agent against them (Figure S3).

Regarding *Acinetobacter* sp. and *Pseudomonas* sp. Isolates, which were the second most predominant species among Gram-negative microbes recovered from ICU patients, the clinical isolates of *Pseudomonas* sp. displayed better sensitivity patterns to ciprofloxacin, levofloxacin, amikacin, and gentamicin than *Acinetobacter* sp., (Table S10 and Figures S4 and S6). By contrast, *Acinetobacter* sp. isolates showed quite better susceptibility to trimethoprim/sulfamethoxazole for different clinical isolates and nitrofurantoin in urine isolates. Although polymyxin B was not active against one urine isolate of *Acinetobacter* sp., it was active against all other strains of *Acinetobacter* sp. and *Pseudomonas* sp. (Table S10).

Most of the Gram-positive bacteria recovered from ICU patients displayed either no or very weak sensitivity to amoxicillin/clavulanate, ampicillin/sulbactam, piperacillin/tazobactam, cefotaxime, ceftazidime, ceftriaxone, and cefepime (Table S11 and Figures S8–S12). Interestingly, many of them showed either good or high susceptibility to ciprofloxacin, levofloxacin, doxycycline, gentamicin, and nitrofurantoin. Similar to other Gram-positive bacteria recovered in our study, the best antibacterial agents evaluated against various ICU Gram-positive microbes were teicoplanin, vancomycin, and linezolid (Figure S8).

As presented in Table S11 and Figure S9, isolates of the coagulase-negative *Staphylococcus* recovered from blood and sputum specimens showed moderate susceptibility to the macrolide antibiotics erythromycin and azithromycin, while teicoplanin, vancomycin, and linezolid, and to less extent, doxycycline exhibited the best antibacterial activity when assessed against many coagulase-negative *Staphylococcus* strains isolated from various clinical samples from ICU patients including urine, blood, and sputum. Similarly, the clinical isolates of *S*. *aureus* recovered from ICU patients as the second most predominant Gram-positive microbe among ICU-retrieved bacteria displayed a somewhat comparable susceptibility pattern to the coagulase-negative *Staphylococcus* (Table S11 and Figure S10). Among the *S. aureus* isolates detected in the ICU, 10 isolates out of 11 tested against cefoxitin were MRSA (91%), with 3 of them recovered from sputum specimens and 1 recovered from urine specimens, as well as 7 isolates obtained from blood cultures, whereas all MRSA isolates showed high sensitivity to vancomycin and linezolid.

Among the isolated Gram-negative bacteria, 7% (11/157) were ESBL-producing organisms, whereas *Klebsiella* sp. was the most predominant ESBL producer, with 12.9% of *Klebsiella* isolates (8/62) having positive production of ESBL. In all, 8% (2/25) of the isolated *E*. *coli* and 33.3% (1/3) of *Proteus* sp. isolates were ESBL-producing microorganisms. Interestingly, 45.4% of the ESBL-producing organisms were isolated from urine specimens followed by sputum specimens (27.2%), wound swabs (18.1%), and blood cultures (9.09%). The highest ESBL producers were isolated from the ICU patients (54.5%) followed by the SUR (27.2%).

Regarding the ESBL-producing organisms, piperacillin/tazobactam, carbapenems, aminoglycosides, and polymyxin B were the most effective antibiotics against the isolates of *E*. *coli*, *Klebsiella* sp., and *Proteus* sp., while levofloxacin and ciprofloxacin were found least efficacious, especially against *Klebsiella* sp.

## 4. Discussion

In this hospital-based analysis of surveillance data from a tertiary care hospital, we surveyed the antibiotic sensitivity profiles of both Gram-positive and Gram-negative nosocomial pathogens isolated from different clinical specimens collected from various healthcare units in the hospital. Our study revealed that the most abundant Gram-negative organisms in all the hospital units were *Klebsiella* sp., *Acinetobacter* sp., and *Pseudomonas* sp., while *Staphylococcus* sp. was the commonest Gram-positive organism. This agrees with the EPIC II study in African hospitals [27]. Further, our study revealed that the emergence of microbial resistance especially among Gram-negative bacteria has been increasing steadily. These data are in line with trends described from different regions all over the world which are considered threatening issues [28,29].

This big increase in antibiotic resistance could be attributed to different reasons, including the inappropriate use of antibiotics which increased recently during the COVID-19 pandemic, as discussed above. Antibiotics were used during the COVID-19 pandemic to treat bacterial infections that coexisted with COVID-19 infections or to take advantage of their potential antiviral effects; however, the relationship between antibiotic resistance and COVID-19 infection was controversial, as several studies suggested that there had been an increase in antibiotic resistance, while the most prevalent bacteria with high antibiotic resistance were *Acinetobacter baumannii* and *Klebsiella pneumonia* from the Gram-negative bacteria in addition to *S. aureus* and *Enterococcus faecalis* from the Gram-positive bacteria [3,6,7], which are quite similar to our findings. However, some other studies assumed that the COVID-19 era did not affect antibiotic resistance among microbes [30].

The highest susceptibility rates recorded in most hospital units for *Klebsiella* sp., *Acinetobacter* sp., and *Pseudomonas* sp. were to polymyxin B, which is truly alarming because polymyxin B is one of the last choices in antibiotic therapy. The constant downward trend in piperacillin/tazobactam, imipenem, meropenem, ciprofloxacin, and levofloxacin activity versus Gram-negative pathogens was one of the most important findings of our research, although in many Gram-negative microorganisms, the susceptibility rates of imipenem and meropenem were slightly better than in piperacillin/tazobactam, ciprofloxacin, and levofloxacin. On the other hand, the susceptibility rate to aminoglycosides such as amikacin and gentamicin was noted to be high in different specimens, which may be due to the smaller frequency of usage due to its nephrotoxicity [31]. Additionally, a high pattern of resistance to ampicillin, amoxicillin, ceftriaxone, cefepime, and ceftazidime was recorded. This is consistent with findings from other studies on the antibiotic resistance of Gram-negative bacilli in critically ill individuals [32,33]. These findings of moderate to high resistance patterns of numerous recovered Gram-negative bacteria from different hospital units to several antibiotics from different antibiotic classes such as penicillins, cephalosporins, carbapenems, penicillins/beta-lactamase inhibitors, and quinolones indicate the growing chance of detecting various antibiotic-degrading enzymes such as the b-lactamases carbapenemase, extended-spectrum beta-lactamases, and metallo-beta-lactamases, among others.

Interestingly, 7% of the Gram-negative bacilli recovered in our study were ESBL producers. In other different studies, the percentage of ESBL generation has been found to range from 17% to 70% [34,35]. However, in the investigation by Sharma et al., ESBL production was shown to be 52.49% [36].

Indeed, *S*. *aureus*, coagulase-negative *Staphylococcus*, and *Enterococcus* species were the most prevalent Gram-positive organisms in our data analysis of this study, while we found a significant sensitivity pattern of most Gram-positive bacteria to linezolid, teicoplanin, and vancomycin. Interestingly, their susceptibility to ciprofloxacin, levofloxacin, doxycycline, gentamicin, erythromycin, azithromycin, clindamycin, and nitrofurantoin has varied between moderate and high among *S*. *aureus* and coagulase-negative *Staphylococcus* isolates, whereas a worse sensitivity pattern was recorded for *Enterococci* isolates. These results agreed with the antibiogram of the Gram-positive isolates in various studies [37,38,39]. It is noteworthy that 14 isolates out of 15 *S*. *aureus* isolates (93%) recovered from different units and tested against cefoxitin were recorded as MRSA, which is an alarming health problem due to limited options for its treatment. By contrast, a previous study conducted by Srikanth et al. found that the MRSA percentage among the coagulase-positive *S. aureus* was 32.2% [40], while another study by Pai et al. found that out of 237 *S*. *aureus* isolates, 69 isolates (29.1%) were recorded as MRSA [41].

In the present study, the antibiogram of our general ICU showed that the Gram-negative organism *Klebsiella* sp. was the most prevalent bacterium in ICU with a percentage of 31%. This high virulence can be related to the fact that these bacteria are part of the normal flora of the human host’s mouth, oropharynx, GI tracts, skin, and intestines [42]. This finding opposes earlier research, notably a study from the United States, which claimed that *Pseudomonas aeruginosa* was the most prevalent bacterium (31.6%) [43] whereas another study in Egypt performed by Elkolaly et al. concluded that *Pseudomonas* sp. was the most prevalent Gram-negative pathogen (37.5%), followed by *Klebsiella* sp. (25%) [44]. However, a recent study in Egypt by Negm et al. revealed that the incidence of *Klebsiella* sp. among recovered pathogens was 33.51% [45].

Most of the *Klebsiella* sp. recovered from the ICU showed low susceptibility to carbapenems, especially in blood cultures (25%), sputum specimens (36%), and wound specimens (27% meropenem and 45% imipenem). This finding is in agreement with the results obtained by Qadeer et al., as carbapenem resistance of *Klebsiella* was 56% for meropenem and 55% for imipenem [46], while in urine specimens, 62% of *Klebsiella* sp. isolates were sensitive to meropenem and 67% to imipenem, which is corroborated by Rajan et al. who documented 28.13% carbapenem resistance of *Klebsiella* [47]. Sheth et al. demonstrated 100% sensitivity of *Klebsiella* to carbapenems [48]. In addition, *Klebsiella* sp. recovered from most specimens displayed a low sensitivity pattern to aminoglycosides, which is consistent with results obtained by Gunjal et al., who reported that 60% of *Klebsiella* were resistant to amikacin, and 80% were resistant to gentamicin [49].

Similarly, clinical isolates of *Acinetobacter* sp. recovered from blood and sputum exhibited no or bad sensitivity to carbapenems, with a percentage of 0% and 11%, respectively, for imipenem and 0% and 12% for meropenem. Our finding matches the findings obtained by Qadeer et al., who found 0% sensitivity of *Acinetobacter* to carbapenems [46]. Other findings by Khan reported 79% resistance of *Acinetobacter* to imipenem [50], whereas Rajan et al. found that 52% of *Acinetobacter* isolates were resistant to carbapenems [47]. Moreover, most *Acinetobacter* isolates recovered from blood, pus, and sputum were not sensitive to aminoglycosides and quinolones. Similarly, Negm and coworkers revealed that *Acinetobacter* was highly resistant to aminoglycosides (82.2% gentamicin and 67.9% amikacin) and fluoroquinolones (91.6% ciprofloxacin and 79.9 levofloxacin) [45].

On the other hand, clinical isolates of *Pseudomonas* Sp. showed moderate to high susceptibility to carbapenems (50–71% for imipenem and 25–86% for meropenem). Similarly, another study conducted by Rakhee et al. showed that 20.8% of *Pseudomonas* isolates were resistant to imipenem [51]. In addition, another previous study performed by Rajan et al. showed a high sensitivity pattern of *Pseudomonas* isolates to carbapenems, as only 12.9% of *Pseudomonas* isolates were resistant to carbapenems 40 [40]. In contrast, Negm et al.’s study revealed that *Pseudomonas* isolates were less sensitive to carbapenems (82.7% resistance to imipenem and 84.7% resistance to meropenem) [45]. Moreover, *Pseudomonas* displayed no or bad sensitivity to most third- and fourth-generation cephalosporins, while they showed moderate to high aminoglycoside susceptibility for most isolates. Radji et al. showed weak sensitivity of *Pseudomonas* isolates to ceftriaxone (60.9% resistance), and amikacin was seen to be the most efficient antibiotic against *Pseudomonas* infections, with only 15.6% resistance [52].

Furthermore, *E. coli* is one of the most prevalent organisms that cause urinary tract infections and it exhibited high sensitivity to carbapenems, with susceptibility rates of 83% for both imipenem and meropenem, confirming the low resistance of *E*. *coli* to carbapenems, as shown previously by Qadeer et al., who reported that only 10% of *E*. *coli* were carbapenem-resistant [46]. Another study reported that 34% of *E*. *coli* isolates were resistant to amikacin. and 53.2% were resistant to gentamicin [44], which is consistent with our results, as 80% of our *E*. *coli* isolates were sensitive to amikacin, while 50% were susceptible to gentamicin.

The most common Gram-positive organism in ICU was the coagulase-negative *Staphylococcus* (14%), which was sensitive to vancomycin and linezolid, with 100% susceptibility. This was followed by *S. aureus* and *Enterococcus*, with a prevalence of 9% and 5% in ICU, respectively. Further, their sensitivity to vancomycin and linezolid was 100%. Interestingly, Savanur et al. found similar results in their previous study. Among Gram-positive microbes, the coagulase-negative *Staphylococci* were the most commonly isolated organisms (15.6%), especially in blood cultures [53]. Indeed, Radha Rani et al. also mentioned that the most prevalent Gram-positive isolates were the coagulase-negative *Staphylococci* (52.80%), followed by *S*. *aureus* (14.40%) and *Enterococcus* species (10.14%) [54], while Chidambaram et al. showed that among Gram-positive isolates, *Enterococcus* was the most frequently recovered bacterium (4.79%), followed by *S. aureus* (3.72%) [55].

In addition, our findings regarding the antibiotic sensitivity pattern of the recovered Gram-positive bacteria were in agreement with earlier studies by Kaur et al., who reported that Gram-positive bacterial isolates showed maximum sensitivity towards vancomycin (100%), whereas the coagulase-negative *Staphylococcus* and *S*. *aureus* were resistant to commonly used antibiotics, including ampicillin, amoxicillin, cloxacillin, and cefoxitin [56]. However, in contrast to our results, Savanur et al. found that the coagulase-negative *Staphylococcus* isolates were 100% resistant to vancomycin [53].

In the present study, we also observed the consequences of local antibiograms among patients undergoing cardiac surgery, postoperative cardiac surgical patients, and those admitted to the CCU. For instance, *Klebsiella*, *Acinetobacter*, and *Pseudomonas* were the most commonly isolated organisms in the CCU and OH. *Klebsiella*, *Acinetobacter*, and *Pseudomonas* isolates recovered from CCU patients showed low or no sensitivity patterns to many tested antibiotics, including ceftazidime, cefotaxime, cefepime, ciprofloxacin, levofloxacin, meropenem, and imipenem. Davoudi et al. reported the incidence of resistance to ceftazidime, ceftriaxone, and ciprofloxacin among *Klebsiella* sp. at 71.42%, 57.1%, and 57.1%, respectively [57].

Additionally, we found that 25% of recovered isolates from patients in the CCU were Gram-positive cocci which were extremely sensitive to vancomycin, teicoplanin, and linezolid, except for *Enterococci* isolates, which were moderately sensitive to teicoplanin (50%). This finding is corroborated by Parameswarappa et al., who showed that *Enterococci* were sensitive to linezolid (98%), vancomycin (64%), and teicoplanin (59.3%) [58]. Gram-positive bacteria may be prevalent in the CCU due to the incidence of cardiac implanted devices, coronary catheterization, and infectious endocarditis [59].

The prevalence results in the SUR showed that the Gram-negative bacteria were the predominant infectious agents in patients admitted to the general surgery unit, while *E. coli* and *Pseudomonas* sp., followed by *Klebsiella* sp., were the most prevalent organisms. *E*. *coli* was found to be less sensitive to ciprofloxacin and levofloxacin, whereas *Pseudomonas* sp. and *Klebsiella* sp. showed low and moderate carbapenem resistance, respectively. Like other hospital units, both *S. aureus* and *Enterococcus* sp. recovered from the SUR were susceptible to vancomycin, linezolid, and teicoplanin, while they were non-susceptible to trimethoprim/sulfamethoxazole, clindamycin, and ampicillin. These findings are consistent with the study on surgical site wound infections in Mulago and Ugandan [60,61].

Lastly, the high frequency of resistance should raise the alarm and highlight the requirement of regular monitoring of the local prevalence of resistance, which could help to determine the most effective antimicrobial treatment and direct experimental therapy.

Given the high, grave, and frightening occurrence of antimicrobial resistance and the restricted availability of empirical antibiotics, a comprehensive effort to battle this enemy should be a top national priority. This program includes infection control policies, care bundles, an antimicrobial stewardship program (ASP), quality, and education. Until the successful deployment of ASP, the only solutions for our ICUs based on the current antibiogram data will be the combination of antibiotics and the provision of newly available drug generations [62]. The present study has some limitations which should be taken into consideration: first, because other bacterial identification systems were required, we were unable to properly identify most of the bacterial isolates at the species level. Second, the use of non-random consecutive sampling techniques may have led to bias in patient recruitment. Third, the fact that this was a single-center study may limit how well our findings generalize to other centers. However, the study methodology can be translated to other hospitals to ascertain their local susceptibilities, and our simulation data can be applied to other units in a more generalized approach.

## 5. Conclusions

Most of the infections at the hospital units in our study led to resistance to most available antibiotics. The last-resort antibiotics, polymyxin B for the Gram-negative bacteria and linezolid for the Gram-positive bacteria, were the only sensitive antibiotics in most hospital-acquired infections. This implies an eventual crisis that threatens the healthcare profession and warrants heightened awareness. Extra legislation is required to prevent healthcare providers from misprescribing and overusing antibiotics, and healthcare providers also need to be better educated on how and when to administer antibiotics in both pandemic and everyday settings.

## Figures and Tables

**Table 1:** Demographic data of included patients.

Parameters	Total (*n* = 221)	ICU (*n* = 143)	CCU (*n* = 24)	OH (*n* = 11)	INT (*n* = 27)	SUR (*n* = 16)
Gender, *n*%						
Male	156 (70.6%)	113 (79.0%)	17 (70.8%)	6 (54.4%)	19 (70.4%)	14 (87.5%)
Female	65 (29.4%)	30 (21.0%)	7 (29.2%)	5 (45.5%)	8 (29.6%)	2 (12.5%)
Age (years), *n*%						
18–39	34 (15.4%)	34 (23.8%)	4 (16.7%)	0 (0%)	6 (22.2%)	2 (12.5%)
40–60	60 (57.1%)	60 (42.0%)	8 (33.3%)	2 (18.2%)	5 (18.5%)	8 (50.0%)
>60	127 (57.5%)	49 (34.3%)	12 (50.0%)	9 (81.8%)	16 (59.3%)	6 (37.5%)
Median (IQR)	65 (49–76.5)	55 (40–64)	47.5 (47–72.5)	74 (68–78)	63 (50–79)	55 (44.25–70.5)
Type of infection, *n*%						
HAP	32 (14.5%)	18 (12.6%)	5 (20.8%)	1 (9.1%)	3 (11.1%)	5 (31.3%)
VAP	24 (10.9%)	13 (9.1%)	5 (20.8%)	3 (27.3%)	1 (3.7%)	2 (12.5%)
UTI	75 (33.9%)	50 (35.0%)	6 (25.0%)	2 (18.2%)	13 (48.1%)	4 (25.0%)
BSI	32 (14.5%)	23 (16.1%)	2 (8.3%)	2 (18.2%)	3 (11.1%)	2 (12.5%)
CRBSI	25 (11.3%)	21 (14.7%)	1 (4.2%)	1 (9.1%)	2 (7.4%)	0 (0%)
SSTI	23 (10.4%)	10 (7.0%)	5 (20.8%)	2 (18.2%)	3 (11.1%)	3 (18.8%)
IAI	10 (4.5%)	8 (5.6%)	0 (0%)	0 (0%)	2 (7.4%)	0 (0%)

IQR: interquartile range, HAP: hospital-acquired pneumonia, VAP: ventilator-associated pneumonia, UTI: urinary tract infection, BSI: bloodstream infection, CRBSI: catheter-related bloodstream infection, IAI: intra-abdominal infection, SSTI: skin and skin soft tissue infection. ICU: intensive care unit, CCU: cardiac care unit, OH: open-heart surgery unit, INT: intermediate care unit, SUR: general surgery unit.

## Data Availability

Available upon request from the corresponding author.

## Supplementary Materials

The following supporting information can be downloaded at: https://www.mdpi.com/article/10.3390/medicina58111597/s1, Figure S1. The total number of different Gram-positive and Gram-negative bacteria recovered from various hospital units, Figure S2. Heatmap of the percentage of antibiotic sensitivity of the Gram-negative isolates recovered from intensive care unit, Figure S3. Antibiotic sensitivity pattern of different isolates of *Klebsiella* sp. recovered from different clinical specimens from intensive care unit, Figure S4. Antibiotic sensitivity pattern of different isolates of *Acinetobacter* sp. recovered from different clinical specimens from intensive care unit, Figure S5. Antibiotic sensitivity pattern of different isolates of *Pseudomonas* sp. recovered from different clinical specimens from intensive care unit, Figure S6. Antibiotic sensitivity pattern of different isolates of *E. coli* recovered from different clinical specimens from intensive care unit, Figure S7. Antibiotic sensitivity pattern of different isolates of *Proteus* sp. recovered from different clinical specimens from intensive care unit, Figure S8. Heatmap of the percentage of antibiotic sensitivity of the Gram-positive isolates recovered from intensive care unit, Figure S9. Antibiotic sensitivity pattern of different isolates of Coagulase-negative Staphylococcus recovered from different clinical specimens from intensive care unit, Figure S10. Antibiotic sensitivity pattern of different isolates of *S. aureus* recovered from different clinical specimens from intensive care unit, Figure S11. Antibiotic sensitivity pattern of different isolates of *Enterococcus* sp. recovered from different clinical specimens from intensive care unit, Figure S12. Antibiotic sensitivity pattern of different isolates of Streptococcus viridans recovered from different clinical specimens from intensive care unit, Table S1. List of antibiotics and their classes used in the antibiotic susceptibility testing of the recovered isolates in the study, Table S2. The total number of recovered bacterial isolates from different hospital units, Table S3. The total numbers of different Gram-positive and Gram-negative bacteria recovered from various hospital units, Table S4. The total numbers of different Gram-positive and Gram-negative bacteria recovered from each hospital unit, Table S5. The total number of different Gram-positive and Gram-negative bacteria recovered from various clinical specimens from patients in intensive care unit, Table S6. Antibiotic sensitivity pattern of different bacterial isolates recovered from the critical cardiac unit in terms of percentage of sensitive isolates, Table S7. Antibiotic sensitivity pattern of different bacterial isolates recovered from the intermediate care unit in terms of percentage of sensitive isolates, Table S8. Antibiotic sensitivity pattern of different bacterial isolates recovered from the open-heart surgery unit in terms of percentage of sensitive isolates, Table S9. Antibiotic sensitivity pattern of different bacterial isolates recovered from the general surgery unit in terms of percentage of sensitive isolates, Table S10. Antibiotic sensitivity pattern of Gram-negative isolates recovered from intensive care unit in terms of percentage of sensitive isolates, Table S11. Antibiotic sensitivity pattern of Gram-positive isolates recovered from intensive care unit in terms of percentage of sensitive isolates.
