# Supplementary material for: Emergence of High Antimicrobial Resistance among Critically Ill Patients with Hospital-Acquired Infections in a Tertiary Care Hospital
Source: Medicina (Kaunas). 2022 Nov 4;58(11):1597. doi: 10.3390/medicina58111597 (PMC9698311; doi:10.3390/medicina58111597)
Supplement: Supplementary file 1 [file medicina-58-01597-s001.zip › medicina-1937525-supplementary.pdf]

Supporting Information

Emergence of High Antimicrobial Resistance among Critically Ill Patients with Hospital-Acquired Infections in a Tertiary Care Hospital

Ahmed E. Abou Warda [1], Fatma Molham[2], Heba F. Salem [3], Gomaa Mostafa-Hedeab[4*], Bashayer F. ALruwaili[5], Ayman N Moharram [6], Mohamed Sebak[2] and Rania M. Sarhan [7]

[1] Clinical Pharmacy Department, Faculty of Pharmacy, October 6 University, Giza, P.O. Box 12585, Egypt, ahmedessamabouwarda@gmail.com

[2] Microbiology and Immunology Department, Faculty of Pharmacy, Beni-Suef University, Beni-Suef 62514, Egypt, fatma.molham@pharm.bsu.edu.eg, mohamed.sebak@pharm.bsu.edu.eg

[3] Pharmaceutics and Industrial Pharmacy Department, Faculty of Pharmacy, Beni Suef University, Beni Suef 62514, Egypt, Heba_salem2004@yahoo.co.uk

[4] Pharmacology department, Medical College, Jouf University, Sakaka 72388, Saudi Arabia.

[5] Community and Family Medicine department, Division of family medicine, Medical College, Jouf University, Sakaka 72388, Saudi Arabia. bfalrwili@ju.edu.sa

[6] Critical Care Medicine Department, Faculty of Medicine, Cairo University, Giza, Egypt, ayman.moharram@kasralainy.edu.eg

[7] Clinical Pharmacy Department, Faculty of Pharmacy, Beni-Suef University, Beni-Suef 62514, Egypt, raniamohammad87@yahoo.com

* Correspondence : E-mail : Gomaa@ju.edu.sa, Tel : +96653462739

<u>**List of Supporting Information:**</u>

Table S1. List of antibiotics and their classes used in the antibiotic susceptibility testing of the recovered isolates in the study.

Table S2. The total number of recovered bacterial isolates from different hospital units.

Table S3. The total numbers of different Gram-positive and Gram-negative bacteria recovered from various hospital units.

Table S4. The total numbers of different Gram-positive and Gram-negative bacteria recovered from each hospital unit.

Table S5. The total number of different Gram-positive and Gram-negative bacteria recovered from various clinical specimens from patients in intensive care unit.

Table S6. Antibiotic sensitivity pattern of different bacterial isolates recovered from the critical cardiac unit in terms of percentage of sensitive isolates.

Table S7. Antibiotic sensitivity pattern of different bacterial isolates recovered from the intermediate care unit in terms of percentage of sensitive isolates.

Table S8. Antibiotic sensitivity pattern of different bacterial isolates recovered from the open-heart surgery unit in terms of percentage of sensitive isolates.

Table S9. Antibiotic sensitivity pattern of different bacterial isolates recovered from the general surgery unit in terms of percentage of sensitive isolates.

Table S10. Antibiotic sensitivity pattern of Gram-negative isolates recovered from intensive care unit in terms of percentage of sensitive isolates.

Table S11. Antibiotic sensitivity pattern of Gram-positive isolates recovered from intensive care unit in terms of percentage of sensitive isolates.

Figure S1. The total number of different Gram-positive and Gram-negative bacteria recovered from various hospital units.

Figure S2. Heatmap of the percentage of antibiotic sensitivity of the Gram-negative isolates recovered from intensive care unit.

Figure S3. Antibiotic sensitivity pattern of different isolates of *Klebsiella* sp. recovered from different clinical specimens from intensive care unit.

Figure S4. Antibiotic sensitivity pattern of different isolates of *Acinetobacter* sp. recovered from different clinical specimens from intensive care unit.

Figure S5. Antibiotic sensitivity pattern of different isolates of *Pseudomonas* sp. recovered from different clinical specimens from intensive care unit.

Figure S6. Antibiotic sensitivity pattern of different isolates of *E. coli* recovered from different clinical specimens from intensive care unit.

Figure S7. Antibiotic sensitivity pattern of different isolates of *Proteus* sp. recovered from different clinical specimens from intensive care unit.

Figure S8. Heatmap of the percentage of antibiotic sensitivity of the Gram-positive isolates recovered from intensive care unit.

Figure S9. Antibiotic sensitivity pattern of different isolates of Coagulase-negative *Staphylococcus* recovered from different clinical specimens from intensive care unit.

Figure S10. Antibiotic sensitivity pattern of different isolates of *S. aureus* recovered from different clinical specimens from intensive care unit.

Figure S11. Antibiotic sensitivity pattern of different isolates of *Enterococcus* sp. recovered from different clinical specimens from intensive care unit.

Figure S12. Antibiotic sensitivity pattern of different isolates of *Streptococcus viridans* recovered from different clinical specimens from intensive care unit.

Table S1. List of antibiotics and their classes used in the antibiotic susceptibility testing of the recovered isolates in the study.

Antibiotic Disc	Class	Abbreviation
**Amoxicillin/clavulanate**	**Aminopenicillins/beta-lactamase inhibitors**	**AMC**
**Ampicillin/Sulbactam**	**Aminopenicillins/ beta-lactamase inhibitors**	**SAM**
**Amikacin**	**Aminoglycosides**	**AMK**
**Azithromycin**	**Macrolides**	**AZM**
**Cefepime**	**4th generation cephalosporins**	**FEP**
**Cefotaxime**	**3rd generation cephalosporins**	**CTX**
**Cefoxitin**	**2nd generation cephalosporins**	**FOX**
**Ceftazidime**	**3rd generation cephalosporins**	**CAZ**
**Ceftriaxone**	**3rd generation cephalosporins**	**CRO**
**Ciprofloxacin**	**Fluoroquinolones**	**CIP**
**Clarithromycin**	**Macrolides**	**CLR**
**Clindamycin**	**Lincosamides**	**CLI**
**Doxycycline**	**Tetracyclines**	**DOX**
**Erythromycin**	**Macrolides**	**ERY**
**Gentamicin**	**Aminoglycosides**	**GEN**
**Imipenem**	**Carbapenems**	**IPM**
**Levofloxacin**	**Fluoroquinolones**	**LVX**
**Linezolid**	**Oxazolidinones**	**LZD**
**Meropenem**	**Carbapenems**	**MEM**
**Nitrofurantoin**	**Nitrofuran antibiotics**	**NIT**
**Piperacillin/tazobactam**	**Penicillins/beta-lactamase inhibitors**	**TZP**
**Polymyxin B**	**Polymyxins**	**PB**
**Trimethoprim/ Sulfamethoxazole**	**Sulfonamides**	**SXT**
**Teicoplanin**	**Glycopeptides**	**TEC**
**Tigecycline**	**Glycylcyclines**	**TGC**
**Vancomycin**	**Glycopeptides**	**VAN**

Table S2. The total number of recovered bacterial isolates from different hospital units. ICU= intensive care unit, INT = intermediate care unit, CCU= cardiac care unit, OH= open-heart surgery unit, SUR= general surgery unit.

ICU	INT	CCU	SUR	OH	Total
143	27	24	16	11	221

Table S3. The total numbers of different Gram-positive and Gram-negative bacteria recovered from various hospital units.

***Acinetobacter* sp.**	***E. coli***	***Klebsiella* sp.**	***Pseudomonas* sp.**	***Proteus* sp.**	***Enterococcus* sp.**	***S. aureus***	**Coagulase-negative *Staphylococcus***	***Streptococcus viridans***	**Non-haemolytic *Streptococcus***	**Total**
**36**	**25**	**62**	**31**	**3**	**19**	**17**	**25**	**2**	**1**	**221**

Table S4. The total numbers of different Gram-positive and Gram-negative bacteria recovered from each hospital unit.

Unit	*Acinetobacter* sp.	*E. coli*	*Klebsiella* sp.	*Pseudomonas* sp.	*Proteus* sp.	*Enterococcus* sp.	*S. aureus*	Coagulase-negative *Staphylococcus*	*Streptococcus viridans*	Non-haemolytic *Streptococcus*
ICU	21	13	45	21	1	7	13	20	2	0
INT	7	5	4	0	0	9	1	0	0	1
CCU	5	3	6	3	1	2	1	3	0	0
SUR	2	4	3	4	1	1	1	0	0	0
OH	1	0	4	3	0	0	1	2	0	0

Table S5. The total number of different Gram-positive and Gram-negative bacteria recovered from various clinical specimens from patients in intensive care unit.

Source	*Acinetobacter* sp.	*E. coli*	*Klebsiella* sp.	*Pseudomonas* sp.	*Proteus* sp.	*Enterococcus* sp.	*S. aureus*	Coagulase-negative *Staphylococcus*	*Streptococcus viridans*	Total
**Blood**	**3**	**1**	**5**	**4**	**0**	**3**	**8**	**17**	**2**	**43**
**Sputum**	**9**	**0**	**11**	**5**	**0**	**0**	**4**	**2**	**0**	**31**
**Urine**	**6**	**12**	**17**	**7**	**1**	**4**	**1**	**1**	**0**	**49**
**Wound/Pus**	**3**	**0**	**12**	**5**	**0**	**0**	**0**	**0**	**0**	**20**
**Total**	**21**	**13**	**45**	**21**	**1**	**7**	**13**	**20**	**2**	**143**

Table S6. Antibiotic sensitivity pattern of different bacterial isolates recovered from the critical cardiac unit (CCU) in terms of percentage of sensitive isolates. * means that not all isolates were tested, while the number between brackets refers to the total number of isolates tested against different antibiotics.

Isolates	No.	AMC	SAM	TZP	FOX	CAZ	CRO	CTX	FEP	CIP	LVX	IPM	MEM	GEN	PB
E. coli	3	0	0* (2)	67	67	0	0	0	50* (2)	67	67	100	100	100	100
Klebsiella sp.	6	17	0* (5)	17	20* (5)	20* (5)	17	17	17	17	17	33	33	33	100
Acinetobacter sp.	5	0	0* (4)	60	50* (4)	25* (4)	0* (2)	0* (4)	20	20	20	50* (4)	50* (4)	75* (4)	100
Pseudomonas sp.	3	0	0	33	0* (2)	0	0	0	33	33	33	33	33	50* (2)	100
Proteus sp.	1	0	0	0	NA	0	0	0	0	0	0	100	100	100	0

Isolates	No.	AMC	SAM	TZP	FOX	CAZ	CRO	CTX	FEP	CIP	LVX	IPM	MEM	GEN	ERY	CLI	TEC	DOX	VAN	LNZ
Enterococcus sp.	2	50	50	0	NA	0	0	0	0	50	50	0* (1)	0* (1)	0	0	0	50	0	100	100
S. aureus	1	0	0	0	0	0	0	0	0	100	100	0	0	NA	0	100	100	100	100	100
Coagulase-negative *Staphylococcus*	3	0	0	0	0	0	0	0	0	100* (2)	100* (2)	NA	NA	100* (2)	33	67	100	100	100	100

Table S7. Antibiotic sensitivity pattern of different bacterial isolates recovered from the intermediate care unit (INT) in terms of percentage of sensitive isolates. * means that not all isolates were tested, while the number between brackets refers to the total number of isolates tested against different antibiotics.

Isolates	No.	AMC	SAM	TZP	FOX	CAZ	CRO	CTX	FEP	CIP	LVX	IPM	MEM	AMK	GEN	SXT	PB					
E. coli	5	0	0* (4)	0	0* (4)	0* (4)	0	0	0	20	0* (4)	40	40	60	100	60	100					
Klebsiella sp.	9	0	0* (8)	33	38* (8)	0* (8)	0* (6)	0* (6)	0* (6)	38* (8)	50* (8)	50* (8)	50* (8)	67	67	38* (8)	100					
Acinetobacter sp.	7	0	0	17* (6)	0* (4)	14	0	0	33* (6)	29	29	29	29	43	71	17* (6)	100					
Pseudomonas sp.	4	0	0	50	50* (2)	0* (3)	0	0	50	100	100	100	100	100	100* (3)	0	100* (3)					
		AMC	SAM	TZP	FOX	CAZ	CRO	CTX	FEP	CIP	LVX	IPM	MEM	AMK	GEN	SXT	ERY	CLI	TEC	DOX	VAN	LNZ
S. aureus	1	0	0	0	0	0	0	0	0	100	100	0	0	NA	100	100	100	100	100	100	100	100
Non-haemolytic Streptococcus	1	0	0	0	0	0	0	0	0	0	0	NA	NA	0	0	0	0	0	100	NA	100	100

Table S8. Antibiotic sensitivity pattern of different bacterial isolates recovered from the open-heart surgery unit (OH) in terms of percentage of sensitive isolates. * means that not all isolates were tested, while the number between brackets refers to the total number of isolates tested against different antibiotics.

Isolates	No.	AMC	SAM	TZP	FOX	CAZ	CTX	FEP	CIP	LVX	IPM	MEM	AMK	GEN	SXT	TGC	PB		
Klebsiella sp.	4	0	0	25	25	0	0* (3)	25	50	50	33* (3)	33* (3)	67* (3)	67* (3)	0	100* (2)	100		
Acinetobacter sp.	1	0	0	0	0	0	0	0	0	0	0	0	0	0	0	0	100		
Pseudomonas sp.	3	0	0	33	0* (2)	0	0	0	67	67	50* (2)	50* (2)	67	100* (2)	0	0	100		
		AMC	SAM	TZP	FOX	CAZ	CTX	FEP	CIP	LVX	IPM	MEM	GEN	SXT	CLI	TEC	DOX	VAN	LNZ
S. aureus	1	0	0	0	0	0	0	NA	0	0	NA	NA	100	0	0	100	0	100	100
Coagulase-negative Staphylococcus	2	0	0	0	0	0	0	0	0	0	0	0	50	0* (1)	50	100	50	100	100

Table S9. Antibiotic sensitivity pattern of different bacterial isolates recovered from the general surgery unit (SUR) in terms of percentage of sensitive isolates. * means that not all isolates were tested, while the number between brackets refers to the total number of isolates tested against different antibiotics.

Isolates	No.	AMC	SAM	TZP	FOX	CAZ	CTX	FEP	CIP	LVX	IPM	MEM	AMK	GEN	SXT	PB
E. coli	4	0	0	100* (3)	75	0	0	25	25	33* (3)	100	100	100* (3)	100	25	100
Klebsiella sp.	3	0	0	50* (2)	33	0	0	0	33	33	33	33	33	33	33	100
Acinetobacter sp.	2	0	0* (1)	50	50	0* (1)	0	0	0	0* (1)	50	50	50	50	0* (1)	100
Pseudomonas sp.	4	0	0	50	0* (2)	0	0	67* (3)	50	50	50	50	50	75	0	100
Proteus sp.	1	0	0	100	100	0	0	NA	100	100	100	100	100	100	0	0

		AMC	SAM	TZP	FOX	CAZ	CTX	FEP	CIP	LVX	AMK	GEN	SXT	CLI	TEC	DOX	VAN	LNZ
Enterococcus sp.	1	0	0	NA	NA	0	0	0	0	0	NA	NA	0	0	100	NA	100	100
S. aureus	1	0	0	0	0	0	0	0	0	0	0	0	0	0	100	0	100	100

Table S10. Antibiotic sensitivity pattern of Gram-negative isolates recovered from intensive care unit (ICU) in terms of percentage of sensitive isolates. * means that not all isolates were tested, while the number between brackets refers to the total number of isolates tested against different antibiotics.

Isolates	No.	AMC	SAM	TZP	FOX	CAZ	CRO	CTX	FEP	CIP	LVX	IPM	MEM	AMK	GEN	SXT	PB	NIT
E. coli (Blood)	1	0	0	100	100	0	0	0	NA	100	100	100	100	100	100	100	100	NA
E. coli (Urine)	12	8	0* (11)	58	60* (10)	9* (11)	10* (10)	8	0* (8)	25* (8)	18* (11)	83	83	80* (10)	50* (10)	0	100* (9)	67
Klebsiella sp. (Blood)	5	0	0* (4)	20	0	0	0	0	0	0	0	25	25	40	40	0	100	NA
Klebsiella sp. (Pus)	1	0	0	0	100	0	0	0	0	100	100	100	100	0	100	0	100	NA
Klebsiella sp. (Sputum)	11	0	0* (10)	20* (10)	10* (10)	10	0	0* (10)	0	18	27	36	36	30* (10)	36	12* (8)	100	NA
Klebsiella sp. (Urine)	17	6	0* (16)	47	43* (15)	0	0* (15)	0* (15)	0* (15)	38* (16)	33* (15)	67* (15)	62* (16)	64* (14)	69* (13)	7* (15)	100* (16)	42* (12)
Klebsiella sp. (Wound)	11	10* (10)	0* (8)	22* (9)	18	10* (10)	0* (9)	10* (10)	11* (9)	10* (10)	10* (10)	45	27	20* (10)	33* (9)	9	100	NA
Acinetobacter sp. (Blood)	3	0	0	0	0	0	0	0	0	0	33	0	0	0	0	33	100	NA
Acinetobacter sp. (Pus)	1	0	0	0	0	0	0	0	0	0	0	100	100	0	0	0	100	NA
Acinetobacter sp. (Sputum)	9	0	0	0	0* (8)	0	0* (6)	0* (8)	11	11	11	11	12* (8)	22	11	11	100	NA
Acinetobacter sp.	6	0	0* (5)	40* (5)	20* (5)	0* (5)	0* (5)	0	17	17	17	83	67	67	67	50* (4)	83	100* (5)

**(Urine)**																		
***Acinetobacter* sp. (Wound)**	**2**	**0**	**0**	**50**	**50**	**0**	**0**	**0**	**50**	**50**	**50**	**100**	**100**	**50**	**50**	**50**	**100**	**NA**
***Pseudomonas* sp. (Blood)**	**4**	**0**	**0**	**25**	**0* (3)**	**25**	**0**	**0**	**0* (3)**	**50**	**50**	**50**	**50**	**50**	**50**	**0**	**100**	**NA**
***Pseudomonas* sp. (Sputum)**	**5**	**0**	**0* (4)**	**0* (4)**	**0* (4)**	**20**	**0* (3)**	**0**	**33* (3)**	**25* (4)**	**20**	**60**	**25* (4)**	**25* (4)**	**40**	**0* (4)**	**100**	**NA**
***Pseudomonas* sp. (Urine)**	**7**	**0**	**0* (4)**	**71**	**0* (6)**	**0* (6)**	**0**	**0**	**0* (5)**	**71**	**71**	**71**	**86**	**17* (6)**	**33* (6)**	**20* (5)**	**100* (6)**	**0* (4)**
***Pseudomonas* sp. (Wound)**	**5**	**0**	**0**	**50* (4)**	**0* (3)**	**20**	**0**	**0* (4)**	**40**	**40**	**40**	**60**	**60**	**80**	**80**	**0**	**100**	**NA**
***Proteus* sp. (Urine)**	**1**	**0**	**NA**	**0**	**0**	**0**	**0**	**0**	**NA**	**100**	**100**	**100**	**100**	**100**	**100**	**100**	**NA**	**0**

Table S11. Antibiotic sensitivity pattern of Gram-positive isolates recovered from intensive care unit (ICU) in terms of percentage of sensitive isolates. * means that not all isolates were tested, while the number between brackets refers to the total number of isolates tested against different antibiotics.

Isolates	No.	AMC	SAM	TZP	FOX	CRO	CAZ	CTX	FEP	CIP	LVX	GEN	CLI	ERY	AZM	TEC	DOX	VAN	LNZ	NIT
Enterococcus sp. (Blood)	3	0	33	0* (1)	NA	NA	0* (2)	0* (2)	50* (2)	67	67	0* (2)	33	0* (2)	0* (2)	100	0	100	100	NA
Enterococcus sp. (Urine)	4	0* (3)	0* (3)	0	0* (2)	0	0	0	0	25	25	0	0	0	0* (1)	100* (3)	0	100	100* (3)	67* (3)
S. aureus (Blood)	8	12	12	12	14* (7)	12	17* (6)	14* (7)	12	50	62	88	62	29* (7)	50* (4)	100	67* (6)	100	100* (7)	NA
S. aureus (Sputum)	4	25	25	0* (3)	0* (3)	0* (3)	0* (3)	0* (3)	0* (3)	25	25	50* (2)	33* (3)	25	33* (3)	100	50	100	100	NA
S. aureus (Urine)	1	0	0	0	0	0	0	0	0	0	0	100	NA	0	0	100	100	100	100	100
Coagulase-negative *Staphylococcus* (Urine)	1	0	0	0	0	0	0	0	0	0	0	0	0	0	0	100	100	100	100	100
Coagulase-negative *Staphylococcus* (Blood)	17	0	0* (15)	6	8* (12)	7* (15)	7* (15)	7* (15)	0* (11)	38* (16)	35	75* (16)	50* (14)	31* (13)	33* (9)	100* (15)	64* (14)	100* (16)	100* (15)	NA
Coagulase-negative *Staphylococcus* (Sputum)	2	0	0	0	0	0	0	0	0	100	100	100	100	50	50	100	100	100	100	NA
Streptococcus viridans (Blood)	2	50	50	50	NA	50	50	50	100* (1)	100	100	100	50	50	NA	100	50	100	100	NA

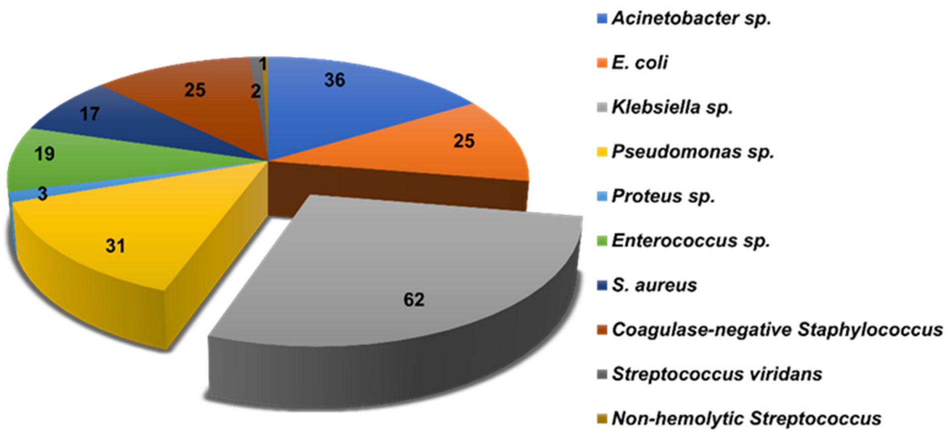

Figure S1. The total number of different Gram-positive and Gram-negative bacteria recovered from various hospital units.

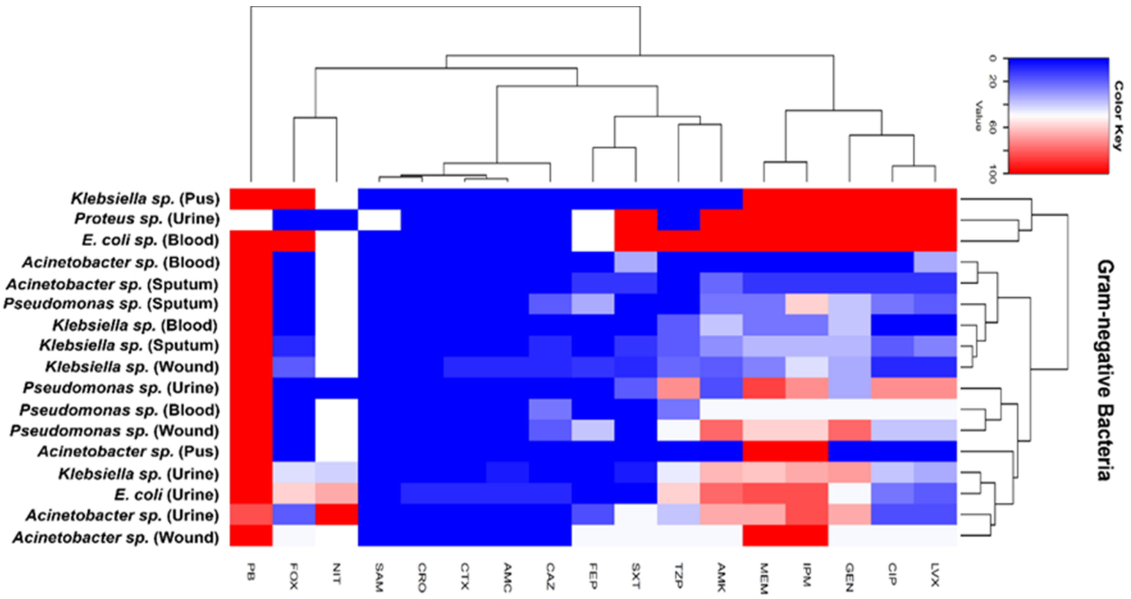

Figure S2. Heatmap of the percentage of antibiotic sensitivity of the Gram-negative isolates recovered from intensive care unit.

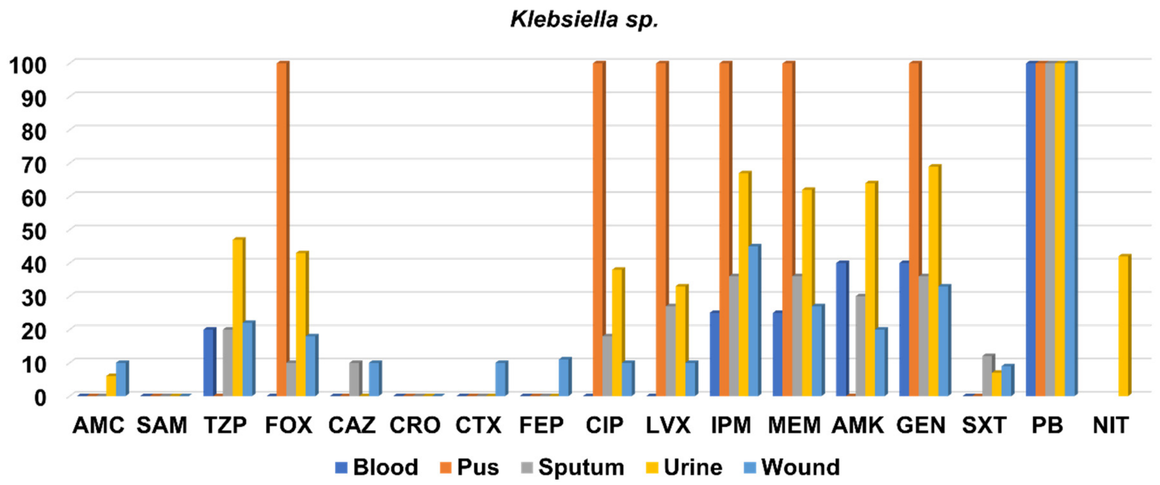

Figure S3. Antibiotic sensitivity pattern of different isolates of *Klebsiella* sp. recovered from different clinical specimens from intensive care unit.

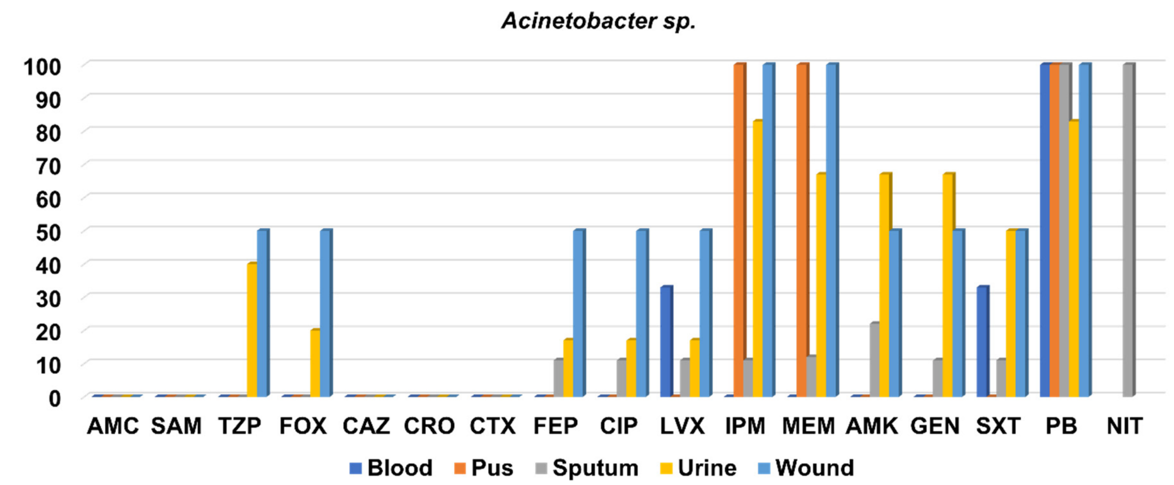

Figure S4. Antibiotic sensitivity pattern of different isolates of *Acinetobacter* sp. recovered from different clinical specimens from intensive care unit.

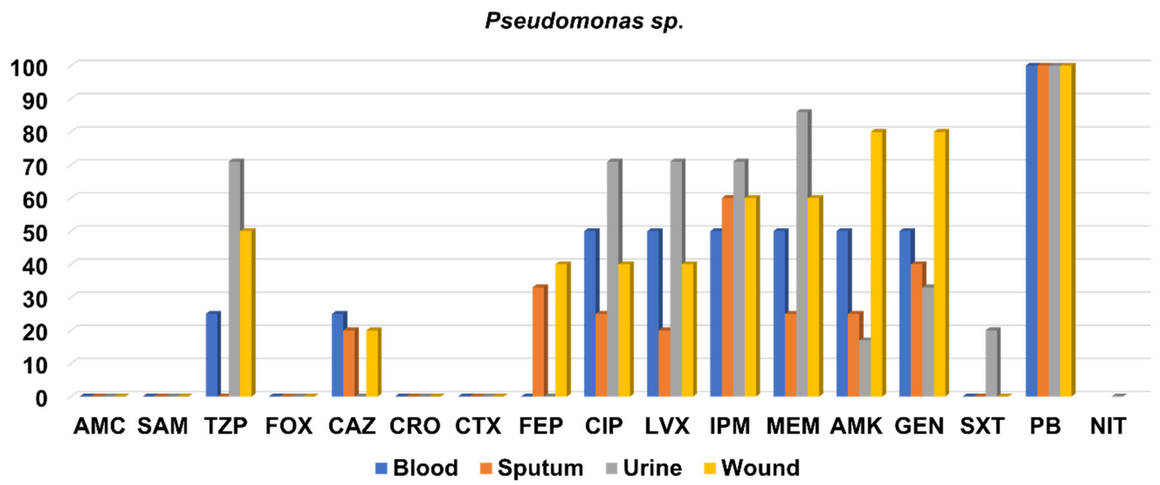

Figure S5. Antibiotic sensitivity pattern of different isolates of *Pseudomonas* sp. recovered from different clinical specimens from intensive care unit.

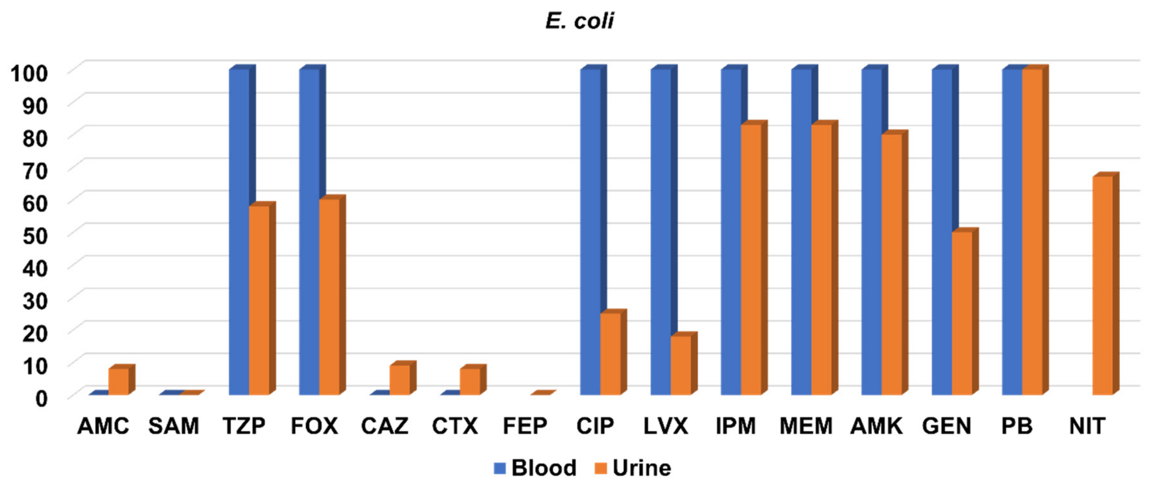

Figure S6. Antibiotic sensitivity pattern of different isolates of *E. coli* recovered from different clinical specimens from intensive care unit.

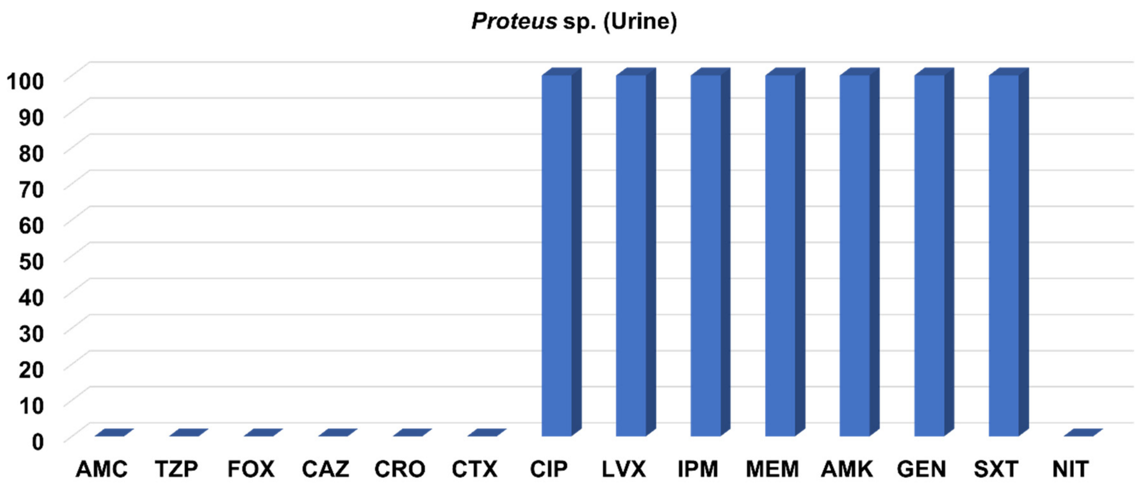

Figure S7. Antibiotic sensitivity pattern of different isolates of *Proteus* sp. recovered from different clinical specimens from intensive care unit.

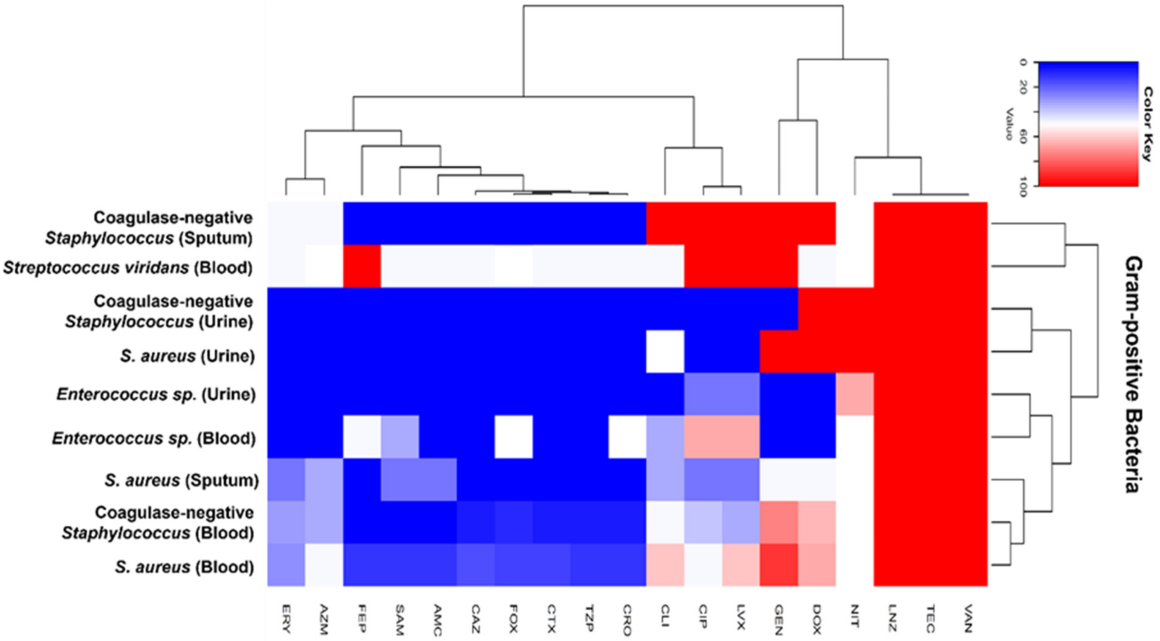

Figure S8. Heatmap of the percentage of antibiotic sensitivity of the Gram-positive isolates recovered from intensive care unit.

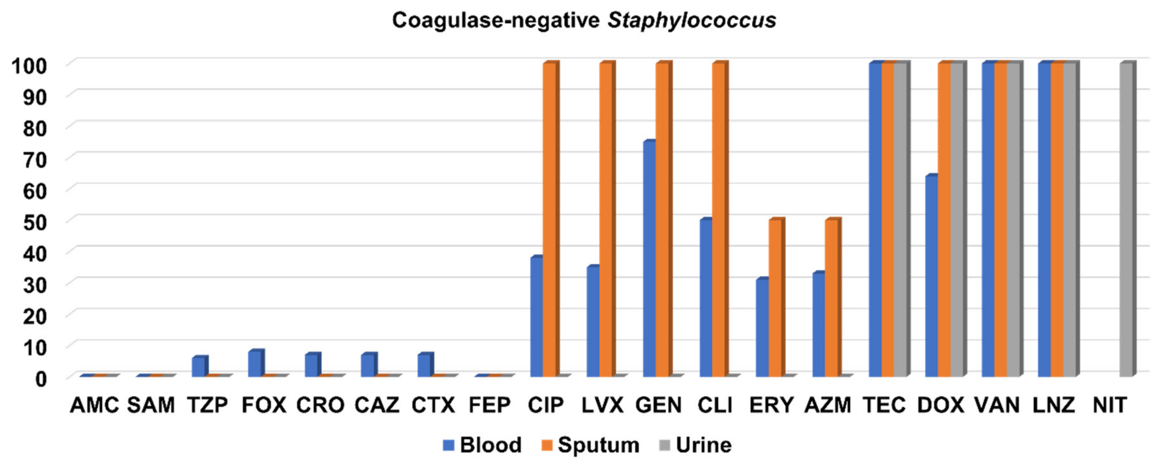

Figure S9. Antibiotic sensitivity pattern of different isolates of Coagulase-negative S*taphylococcus* recovered from different clinical specimens from intensive care unit.

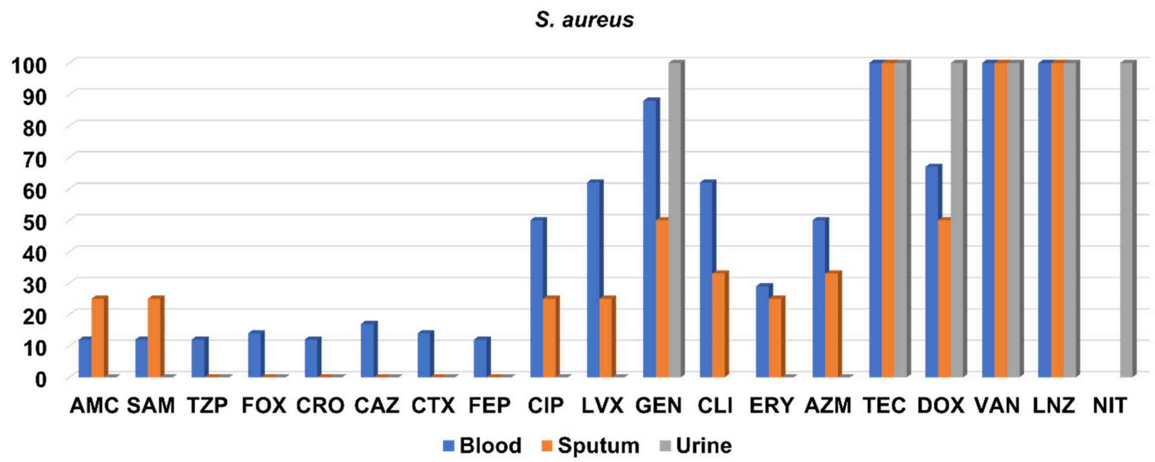

Figure S10. Antibiotic sensitivity pattern of different isolates of *S. aureus* recovered from different clinical specimens from intensive care unit.

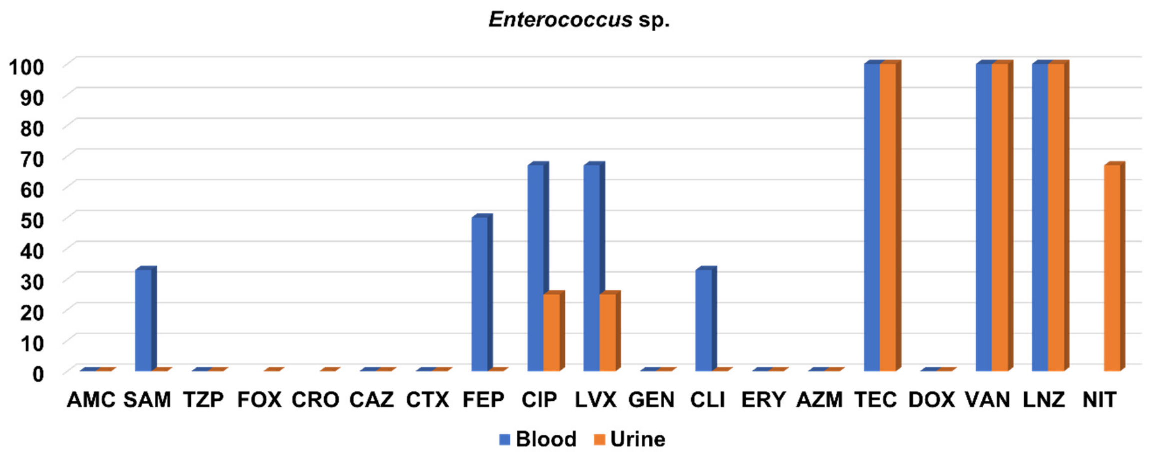

Figure S11. Antibiotic sensitivity pattern of different isolates of *Enterococcus* sp. recovered from different clinical specimens from intensive care unit.

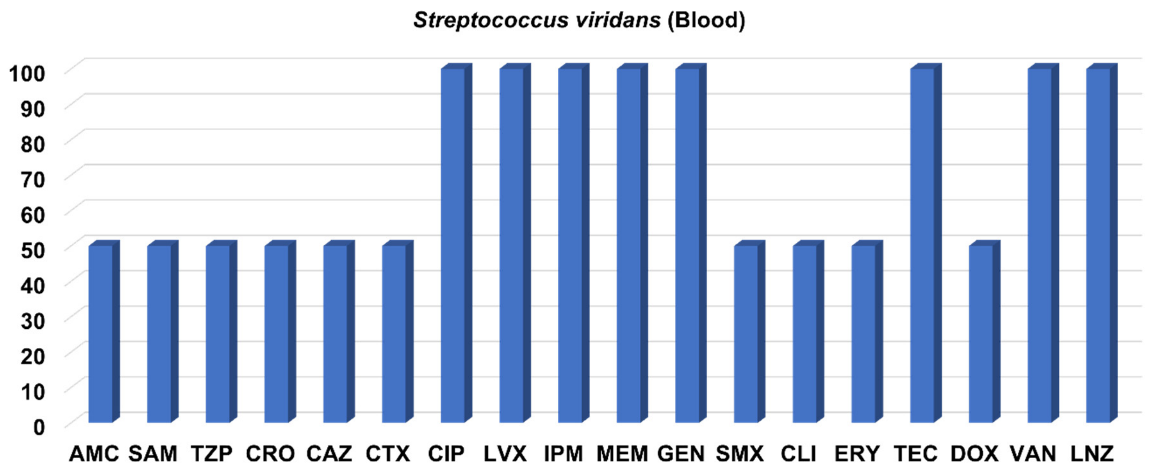

Figure S12. Antibiotic sensitivity pattern of different isolates of *Streptococcus viridans* recovered from different clinical specimens from intensive care unit.
